# Genetic Variations rs11892031 and rs401681 Are Associated with Bladder Cancer Risk in a Chinese Population

**DOI:** 10.3390/ijms151119330

**Published:** 2014-10-24

**Authors:** Yu Zhang, Yan Sun, Tao Chen, Hailong Hu, Wanqin Xie, Zhihui Qiao, Na Ding, Linguo Xie, Sheng Li, Wenlong Wang, Chen Xing, Yihan Wang, Yunkai Qie, Changli Wu

**Affiliations:** 1 Department of Urology, the Second Hospital of Tianjin Medical University, Pingjiang Road 23, Hexi District, Tianjin 300211, China; E-Mails: xielinguo@tijmu.edu.cn (Y.Z.); chentao@tmu.edu.cn (T.C.); hhllove2004@163.com (H.H.); qiaozh2003@126.com (Z.Q.); xielinguo@126.com (L.X.); lishengwudi1250@163.com (S.L.); wangwl1989@126.com (W.W.); xc2772@163.com (C.X.); wangyih1988@126.com (Y.W.); yunkaiqie@sohu.com (Y.Q.); 2 Tianjin Key Laboratory of Urology, Tianjin Institute of Urology, the Second Hospital of Tianjin Medical University, Tianjin 300211, China; E-Mails: sungravel@139.com (Y.S.); wanqinxie@126.com (W.X.); dingnalove2003@126.com (N.D.)

**Keywords:** bladder cancer, genetic variations, polymorphism, genome-wide association studies

## Abstract

Genome-wide association studies (GWAS) have identified a number of genetic variants associated with risk of bladder cancer in populations of European descent. Here, we assessed association of two of these variants, rs11892031 (2q37.1 region) and rs401681 (5p15.33 region) in a Chinese case-control study, which included 367 bladder cancer cases and 420 controls. We found that the AC genotype of rs11892031 was associated with remarkably decreased risk of bladder cancer (adjusted odds ratio (OR), 0.27; 95% confidence interval (CI), 0.09–0.81; *p* = 0.019), compared with the AA genotype of rs11892031; and that CT/CC genotypes of rs401681 were associated with significantly increased risk of bladder cancer (adjusted OR, 1.79; 95% CI, 1.10–2.91; *p* = 0.02), compared with the TT genotype of rs401681. We further conducted stratification analysis to examine the correlation between single nucleotide polymorphism (SNP) rs11892031/rs401681 and tumor grade/stage. Results showed that heterogeneity in ORs of tumor categories was not significant for either rs11892031 or rs401681 (*p* > 0.05), indicating that the two SNPs seemingly do not associate with tumor grade and stage of bladder cancer in our study population. The present study suggests that the SNPs rs11892031 and rs401681 are associated with bladder cancer risk in a Chinese population. Future analyses will be conducted with more participants recruited in a case-control study.

## 1. Introduction

Bladder cancer is one of the most common malignant neoplasms in the world [1]; according to data from 2012 provided by the International Agency for Research on Cancer [2], 55,486 cases and 26,820 deaths were estimated for the population in China, which indicates an incidence rate of 3.0/100,000 and accounts for a large fraction of bladder cancer in East Asia (85,451 cases and 37,491 deaths). In developed countries, like the United States (US) and the European Union (EU), whose populations are predominantly Caucasian, 68,639 new cases and 16,468 deaths were estimated for the US population, and 124,188 new cases and 40,619 deaths for the EU population. The bladder cancer incidence rate in the EU and US was 10.8/100,000 and 11.6/100,000, respectively, much higher than those in China and East Asia (3.6/100,000). However, it is of note that the incidence of bladder cancer has been steadily increasing in China over recent years [3].

Histological types of bladder cancer include urothelial cell carcinoma, which accounts for 95% of bladder cancer cases, as well as adenocarcinoma, squamous cell carcinoma, and other rare types [4,5]. Urothelial cell carcinoma could be in a complex with the other histological variants, which influence the prognosis [6]. About 75% of patients are diagnosed with non-muscle-invasive bladder cancer (NMIBC), corresponding to the clinical stages Ta, T1 and CIS (carcinoma in situ), which generally have a good prognosis, though require repeated cystoscopic evaluations due to frequent local recurrences. The rest of the patients are usually diagnosed with muscle-invasive bladder cancer (MIBC) (stage T2–T4), which is characterized by the invasion into at least the smooth muscle layer (muscularis propria) and is associated with high risk of metastasis. Currently, despite that a variety of histopathologic manifestations have provided some useful information and been applied to the evaluation of prognosis, it is still a great challenge to provide accurate predictions for individual patients [7].

Cigarette smoking and occupational exposure to specific carcinogens are major risk factors for urinary bladder cancer (UBC). Direct exposure to the urine, in which carcinogens such as aromatic amines found in cigarette smoke are enriched, may initiate oncogenic transformation of bladder epithelium, eventually leading to cancer [8]. Familial clustering of UBC cases suggests that genetic variants may play a role in bladder carcinogenesis [9,10,11,12]. In accordance with this, genome-wide association studies (GWAS) have identified a number of genetic variants associated with risk of bladder cancer in populations of European descent [13,14,15,16,17,18,19]. These identified single nucleotide polymorphisms (SNPs) have also been examined in a Chinese population [20,21,22,23,24,25]. Due to the fact that the incidence of bladder cancer differs greatly in countries and areas [3,26,27], it is worthwhile to validate bladder cancer risk-associated SNPs in other independent case-control series. Our initial efforts focused on the two SNPs rs1189031 and rs401681. The SNP rs11892031 is located in an intronic region of the *UDP glucuronosyltransferase 1A* (*UGT1A*) gene, which is implicated in detoxification of many carcinogens. The expression level of *UGT1A* may affect the removal of carcinogens from bladder epithelium [8]. The SNP rs401681 has been reported to be associated with many cancer types, including bladder cancer [16,28,29,30].

In the present study, we analyzed the two SNPs, rs11892031 and rs401681, to evaluate their associations with UBC and with the tumor grade and clinical stage of bladder cancer in a case-control study of a Chinese population.

## 2. Results and Discussion

### 2.1. The Characteristics of Patients and Controls

The key demographic and clinical information for 367 cases and 420 controls are presented in Table 1. There was no significant difference in the mean age between cases and controls (65.9 years *versus* 65.0 years; two-sided *t*-test, *p* = 0.305). No significant difference in age or sex distribution was observed between cases and controls (χ^2^ test, *p* = 0.752 and *p* = 0.451, respectively). However, the distribution of smokers in cases was significantly different from that in controls (χ^2^ test, *p* < 0.001), with the cases having much higher percentage of former smokers (31.41%) than the controls (15.95%) and consequently less never smokers than the controls. Considering that age, sex and smoking status may affect the results, these variables were used for adjustment in the multivariate logistic regression analysis. The genotype distributions for SNP rs11892031 and rs401681 in controls were in accordance with the predicted from the Hardy–Weinberg equilibrium model (*p* > 0.05).

**Table 1:** Characteristics of the study population

Variables	Cases (*n* = 367)	Controls (*n* = 420)	*p*
	*n* (%)	*n* (%)	
**Age (years)**			
Mean ± SD	65.9 ± 11.8	65.0 ± 11.7	0.305
≤65	168 (45.78)	197 (46.90)	0.752
>65	199 (54.22)	223 (53.10)	
**Sex**			
Female	69 (18.80)	88 (20.95)	0.451
Male	298 (82.20)	332 (79.05)	
**Smoking Status**			
Never smoker	149 (40.60)	252 (60.00)	<0.001
Former smoker	115 (31.34)	67 (15.95)	
Current smoker	103 (28.06)	101 (24.05)	
**Tumor Grade**			
Low grade	135 (36.78)	–	–
High grade	232 (63.22)	–	–
**Tumor Stage**			
NMIBC	276 (75.20)	–	–
MIBC	91 (24.80)	–	–

Two-sided *t*-test for categorical variables and Student’s *t*-test for continuous variables; Low grade contains papillary urothelial neoplasm of low malignant potential (PUNLMP) and low grade urothelial carcinoma; NMIBC: non-muscle-invasive bladder cancer; MIBC: muscle invasive bladder cancer.

### 2.2. Association between rs11892031 and Bladder Cancer Susceptibility

The allele and genotype frequencies of rs11892031 for the cases and controls are presented in Table 2. The CC genotype was not obtained in the present study. Our analysis showed that the AC genotype was associated with decreased bladder cancer risk (adjusted OR, 0.27; 95% confidence interval (CI), 0.09–0.81; *p* = 0.019) when the AA genotype served as reference. We further assessed the association between rs11892031 and tumor grade and stage of bladder cancer. As shown in Table 3, the AC genotype showed the direction of association with decreased risk in high-grade bladder cancer (adjusted OR, 0.20; 95% CI, 0.05–0.88; *p* = 0.034) and NMIBC (pTa–pT1) (adjusted OR, 0.27; 95% CI, 0.08–0.93; *p* = 0.038). There was no significant association between the AC genotype and low-grade bladder cancer (adjusted OR, 0.39; 95% CI, 0.09–1.73; *p* = 0.218), nor between the AC genotype and MIBC (adjusted OR, 0.26; 95% CI, 0.03–1.99; *p* = 0.195). Taken together, no heterogeneity in ORs between tumor grades or stages was observed for rs11892031 (*p* > 0.05), indicating that rs11892031 did not associate with bladder tumor grade and stage in this study population.

**Table 2:** Genotype and allele frequencies of the rs11892031 polymorphism among cases and controls and their associations with risk of bladder cancer.

Genotypes	Cases (*n* = 367)	Controls (*n* = 420)	*p*	OR (95% CI) *
	*n* (%)	*n* (%)		
AA	363 (98.91)	401 (95.48)	–	1.00
AC	4 (1.09)	19 (4.52)	0.019	0.27(0.09–0.81)
CC	–	–	–	–
A allele	0.995	0.977	–	1.00
C allele	0.005	0.023	0.020	0.28(0.09–0.82)

* Adjusted for age, sex and smoking status in logistic regression model.

**Table 3:** Stratification analysis of association between rs11892031 polymorphism and bladder cancer.

Variables	rs11892031 Genotype, *n* (%)		*p*	OR (95% CI) *
	AA	AC		AC *versus* AA
Controls (*n* = 420)	401 (95.48)	19 (4.52)	–	1.00
**Cases (*n* = 367)**				
Total	363 (98.91)	4 (1.09)	0.019	0.27 (0.09–0.81)
**Tumor Grade**				
Low grade	133 (98.51)	2 (1.49)	0.218	0.39 (0.09–1.73)
High grade	230 (99.14)	2 (0.86)	0.034	0.20 (0.05–0.88)
**Tumor Stage**				
NMIBC	273 (98.91)	3 (1.09)	0.038	0.27 (0.08–0.93)
MIBC	90 (98.90)	1 (1.10)	0.195	0.26 (0.03–1.99)

* Adjusted for age, sex and smoking status in logistic regression model. Abbreviations: CI, confidence interval.

### 2.3. Association between rs401681 and Bladder Cancer Susceptibility

The allele and genotype frequencies of rs401681 for the cases and controls are presented in Table 4. We found that both the CT (adjusted OR, 1.69; 95% CI, 1.01–2.81; *p* = 0.044) and CC genotype (adjusted OR, 1.90; 95% CI, 1.13–3.18; *p* = 0.015) were associated with increased bladder cancer risk. Additionally, considering the association of rs401681 C allele with bladder cancer and the relatively small result of CT genotype, compared with TT genotype (adjusted OR, 1.69; 95% CI, 1.01–2.81; *p* = 0.044 for CT genotype), we combined CT and CC genotypes as a dominant genetic model in the subsequent analysis. The combined group had a significant association with increased bladder cancer risk as well (adjusted OR, 1.79; 95% CI, 1.10–2.91; *p* = 0.020). Furthermore, as shown in Table 5, C allele carriers were prone to have a significant increase in risk of high-grade bladder cancer (adjusted OR, 2.24; 95% CI, 1.21–4.14; *p* = 0.011) and a considerable increase in risk of MIBC (pT2–pT4) (adjusted OR, 3.21; 95% CI, 1.13–9.16; *p* = 0.029), as reflected by *p* value. There was no significant association observed between the CT/CC genotypes and low-grade bladder cancer (adjusted OR, 1.24; 95% CI, 0.66–2.31; *p* = 0.503), and nor between the CT/CC genotypes and NMIBC (adjusted OR, 1.59; 95% CI, 0.95–2.66; *p* = 0.078). Similar to the case of rs11892031, no heterogeneity in ORs between the bladder cancer categories was detected for rs401681 (*p* > 0.05). Thus, the association of rs401681 with bladder tumor grade and stage was not supported in our case-control study population.

**Table 4:** Genotype and allele frequencies of the rs401681 polymorphism among cases and controls and their associations with risk of bladder cancer.

Genotypes	Cases (*n* = 367)	Controls (*n* = 420)	*p*	OR (95% CI) *
	*n* (%)	*n* (%)		
TT	28 (7.63)	53 (12.62)	–	1.00
CT	166 (45.23)	187 (44.52)	0.044	1.69 (1.01–2.81)
CC	173 (47.14)	180 (42.86)	0.015	1.90 (1.13–3.18)
CT/CC	339 (92.37)	367 (87.38)	0.020	1.79 (1.10–2.91)
T allele	0.302	0.349	–	1.00
C allele	0.698	0.651	0.035	1.26 (1.02–1.57)

* Adjusted for age, sex and smoking status in logistic regression model.

**Table 5:** Stratification analysis of association between rs401681 polymorphism and bladder cancer.

Variables	rs401681 Genotype, *n* (%)		*p*	OR (95% CI) *
	TT	CT/CC		CT/CC *versus* TT
Controls (*n* = 420)	53 (12.62)	367 (87.38)	–	1.00
**Cases (*n* = 367)**				
Total	28 (7.63)	339 (92.37)	0.020	1.79 (1.10–2.91)
**Tumor grade**				
Low grade	14 (10.37)	121 (89.63)	0.503	1.24 (0.66–2.31)
High grade	14 (6.03)	218 (93.97)	0.011	2.24 (1.21–4.14)
**Tumor stage**				
NMIBC	23 (8.33)	253 (91.67)	0.078	1.59 (0.95–2.66)
MIBC	4 (4.40)	87 (95.60)	0.029	3.21 (1.13–9.16)

* Adjusted for age, sex and smoking status in logistic regression model.

### 2.4. Discussion

To our knowledge, although a number of bladder cancer risk-associated loci previously identified and confirmed in Europeans have been verified in a Chinese population [20,21,22,23,24,25], the two SNPs rs1189031 and rs401681 are waiting to be verified. Our study was to verify the association of the two SNPs with bladder cancer in a Chinese population, and assess these two variants in the relationship with tumor grade and stage of bladder cancer. We have demonstrated here that the two susceptibility loci are statistically associated with bladder cancer risk in our Chinese case-control study population. However, rs11902031 and rs401681 showed no statistical significance in the stratification analysis for the tumor grade and stage of bladder cancer.

The SNP rs11892031 is located in an intronic region of the *UDP-glucuronosyltransferase 1A* (*UGT1A*) gene locus. The *UGT1A* gene plays a role in detoxification of many endogenous (steroids, bilirubin) and xenobiotic (drugs, carcinogens) substrates, mainly in the liver but also in extrahepatic tissues [31]. The *UGT1A* locus encodes at least nine *UDP-glucuronosyltransferase1A* isoforms, which are formed by differential splicing [32]. Rothman and colleagues have associated rs11892031 in the *UGT1A* locus on chromosome 2q37.1 with urinary bladder cancer risk in a recent genome-wide association study of European descent [16]. In the present study, we showed that the AC genotype of rs11892031 was associated with decreased bladder cancer risk in a Chinese population, compared with the AA genotype, suggesting that the rs11892031 C allele has a protective effect on bladder cancer risk; our finding is consistent with that in the study by Rothman *et al.* Selinski and colleagues showed that the A allele of rs11892031 was associated with increased bladder cancer risk [32]. Recently, Ma and colleagues conducted a study to investigate the association between rs11892031 and bladder cancer in a Chinese population [23]. Although not reaching statistical significance, the SNP showed the same direction of association with the reported “A” risk allele in Caucasians. Additionally, Tang and colleagues reported the identification of SNP rs17863783 of a functional splicing form, *UGT1A6.1*, within the *UGT1A* gene cluster. *UGT1A6.1* protein may protect from bladder cancer by increasing the removal of carcinogens from bladder epithelium [8]. This work explained and supported the association of SNPs of *UGT1A* region with bladder cancer. In our study, the CC genotype was not obtained, which may be due to the sample size and the rareness of C allele for rs11892031. It was shown that the frequency of rs11892031 A allele in European controls was 92%, while in Asian controls it was 96% [23]. In the present study, the frequency of rs11892031 A allele in control group is 97.7%, close to that in Asian controls. This suggests that the allele frequency of rs11892031 is quite different among different ethnicities. Furthermore, stratification analysis was conducted for tumor grade and stage of bladder cancer with adjustment for age, sex, and smoking status. However, we found no evidence for association of genetic variant rs11892031 with the stratification of bladder cancer, including the tumor grade and stage (*p*_heterogeneity_ > 0.05) in our study population. Considering that our sample size in subgroups was relatively small and the rareness of C allele for rs11892031, this finding should be interpreted with caution and be validated in future, larger studies in Chinese populations.

Following their GWAS composed of 20,726 cancer patients and 134,650 controls, Rafnar and colleagues found that the C allele of rs401681 on chromosome 5p15.33 was associated with bladder cancer risk with a combined OR of 1.12 (95% CI, 1.03–1.11) for the nine European case-control groups tested [28]. GWAS conducted by Rothman and colleagues, with 7673 cases and 40,105 controls of European descent, showed the association between the C allele of rs401681 and bladder cancer risk [16]. Recently, Ma *et al.* examined the association between SNP rs401681 and bladder cancer risk in a Chinese population of 184 cases and 962 controls. However, it did not reach statistical significance (OR, 1.02; 95% CI, 0.80–1.29; *p* = 0.88) [23]. In the present study, we demonstrated the association between rs401681 polymorphism and bladder cancer susceptibility in a northern Chinese population (Tianjin) (adjusted OR, 1.79; 95% CI, 1.10–2.91; *p* = 0.020). In addition, we found that the risk allele frequency of rs401681 was also quite different among different ethnicities. The frequency of rs401681 C allele in European controls was 53.5%, while in Asian controls it was 67.0% [23]. The frequency of rs401681 C allele in our control group is 65.1%, close to that in Asian controls.

Chromosome 5p15.33 region contains the *CLPTM1L* (cleft lip and palate transmembrane 1 like gene) and *TERT* (telomerase reverse transcriptase) genes and genetic variations in this region have been associated with increased or decreased risk of multiple cancer types [29,30]. Rs401681 is located in an intron of the *CLPTM1L* [30]. Although the function of the *CLPTM1L* is largely unknown, it has been shown to influence the sensitivity of ovarian cancer cells to cisplatin-induced apoptosis [33]. A study by James and colleagues showed that *CLPTM1L* was frequently over-expressed in lung cancer and played an anti-apoptotic role by protecting cancer cells from genotoxic-stress induced DNA damage [34]. Apart from the involvement in ovarian and lung cancer, the SNP rs401681 on chromosome 5p15.33 was frequently identified to be associated with increased bladder cancer risk, suggesting its important role in cancer etiology [16,28]. Considering that smoking is a major risk factor for developing bladder cancer, it is possible that the risk variants identified at the 5p15 locus affect the *CLPTM1L* gene, thereby influencing the metabolism of carcinogens, and hence modify cancer risk [4]. Additionally, Rothman *et al.* showed that the C allele carriers of rs401681 had a significantly increased risk in G1 cases (OR, 1.22; 95% CI, 1.09–1.36) and bladder cancer with low risk of progression (stage Ta, low grade) (OR, 1.23; 95% CI, 1.11–1.36). In the present study, our findings were inconsistent with those of Rothman *et al.* [16]. Heterogeneity in ORs for the rs401681 polymorphism between tumor grades or stages was not statistically significant (*p* > 0.05), which did not support an association of rs401681 with tumor grade/stage for bladder cancer. This discrepancy may be due to the genetic heterogeneity between ethnic groups and difference in sample sizes.

## 3. Experimental Section

### 3.1. Patients and Controls

All subjects were of Chinese Han descent. Three-hundred and sixty-seven bladder cancer patients were selected from the Second Hospital of Tianjin Medical University, Tianjin, northern China. All cases were patients newly diagnosed with histologically confirmed transitional cell carcinoma of bladder. The exclusion factors included previous cancer, metastasized cancer from other or unknown origin, and previous radiotherapy or chemotherapy. As a control group, 420 sex- and age-matched healthy subjects without previous history of cancer were recruited to the same hospital during the same period of time. The exclusion criteria for the controls were significant mental impairment or blood transfusion in the past months; controls were also excluded if they had symptoms suggestive of bladder cancer, such as hematuria. Epidemiologic and demographic data were collected through in-person interviews. According to the criteria described by the US Centers for Disease Control and Prevention [35], subjects that never smoked cigarettes or smoked fewer than 100 cigarettes in their entire life were defined as never smokers; Those who smoked at least 100 cigarettes in their entire life but were not currently smoking were defined as former smokers, and the other smokers who had smoked at least 100 cigarettes in their entire life and were still smoking were considered as current smokers. A 4 mL peripheral venous blood sample was obtained for each subject after the interview. All subjects in the present study were provided written informed consent before the collection of epidemiological data and blood samples. The present study was approved by the medical ethics committee of The Second Hospital of Tianjin Medical University (Tianjin, China; Identification code: 201212132; 13 December 2012).

### 3.2. Classification of Grade and Stage for Tumor

Pathological diagnosis for tumor stage in all cases followed the 2002 International Union Against Cancer (UICC) tumor-nodes-metastasis classification and for grading it referenced the World Health Organization (WHO)/International Society of Urological Pathology (ISUP) 2004 grading of urothelial papilloma. Tumor grades stratification refers to either high grade or low grade. In addition, considering papillary urothelial neoplasm of low malignant potential (PUNLMP) characterized by a substantial incidence of recurrence and progression [36], we classified PUNLMP as low grade. Meanwhile, for tumor stage, we classified bladder cancer patients into NMIBC (pTa–pT1 stage) and MIBC (pT2–pT4 stage).

### 3.3. Genotyping

Genomic DNA was isolated from venous blood of patients and controls by standard methods. Genomic DNA was genotyped using polymerase chain reaction–ligation detection reaction (PCR–LDR) method. The sequences of specific primers for multiplex polymerase chain reaction (PCR) are listed as follows:

Rs11892031 forward: 5'-GGTTTGAGGTCTTGTGTGTAG-3', reverse: 5'-GTCTCCCACAACAAGTGAATC-3'; rs401681 forward: 5'-AGGAAACCAAAGGCATAGACC-3', reverse: 5'-CTCCAAAGTTGTCGTAGACTC-3'.

After the amplification, we performed a second-round PCR; the reaction mixture consisted of the following components: 1 μL genome DNA, 1.5 μL Buffer, 1.5 μL MgCL_2_, 0.3 μL dNTP, 0.3 μL Taq DNA polymerase, 9.8 μL H_2_O, 0.3 μL Primer mix. The amplification procedure consisted of initial denaturation at 94 °C for 3 min, 35 cycles of denaturation at 94 °C for 15 s, annealing at 55 °C for 15 s and extension at 72 °C for 30 s, followed by a final extension at 72 °C for 3 min. The ligation reaction was carried out in a final volume of 10 μL containing 1 μL Taq DNA ligase (Generay, Shanghai, China), 0.03 μL Prob mix, 3 μL of Multi-PCR product, 5.845 μL H_2_O, 0.125 μL Taq DNA ligase. The LDR was performed using 30 cycles of denaturation at 95 °C for 3 min, annealing at 94 °C for 30 s and extension at 56 °C for 3 min. The LDR fluorescent product was analyzed using an ABI 3730xl genetic analyzer (Applied Biosystems, Foster City, CA, USA) and the results were analyzed with GeneMapper software (Applied Biosystems). The assays of genotyping were carried out by two people, independently, in a blind fashion. Ten percent of masked samples were randomly selected for confirmation with direct sequencing, and the results were 100% concordant.

### 3.4. Statistical Analysis

Hardy-Weinberg equilibrium (HWE) was carried out by a goodness-of-test for the distribution of genotypic frequencies among the controls. Differences between cases and controls in demographic characteristics, risk factors and frequencies of allele and genotype of each SNP were evaluated by two-sided test (for categorical variables) or Student’s *t*-test (for continuous variables). After adjustment of age, sex, and smoking status, unconditional univariate and multivariate logistic regression was applied to calculate odds ratios (ORs) and 95% confidence intervals (CIs) to estimate the association between the polymorphism and susceptibility of bladder cancer. The χ^2^-based Q-test was used to test the heterogeneity of effect sizes between subgroups. Heterogeneity between ORs was considered to be significant when *p* < 0.05. All analyses were performed using SPSS 20.0 software (IBM, Armonk, NY, USA). All statistical tests were two sided and *p* < 0.05 was considered statistically significant.

## 4. Conclusions

In conclusion, we have demonstrated that the rs11892031 and rs401681 polymorphisms are associated with bladder cancer risk in a Chinese population, suggesting that the two variants may be a potential diagnostic marker for genetic susceptibility of bladder cancer. Certainly, there are a couple of limitations that should be kept in mind for the present study. First, the sample size might affect the power to detect the association between the two variations and bladder cancer. Second, our study is a hospital-based case–control study, therefore we could not exclude the chance of selection bias. In future, further investigation in larger populations and functional characterizations will be conducted to validate our findings.
